# *Brucella suis* Infection in Dog Fed Raw Meat, the Netherlands

**DOI:** 10.3201/eid2406.171887

**Published:** 2018-06

**Authors:** Marloes A.M. van Dijk, Marc Y. Engelsma, Vanessa X.N. Visser, Marcel A.H. Spierenburg, Marjolijn E. Holtslag, Peter T.J. Willemsen, Jaap A. Wagenaar, Els M. Broens, Hendrik I.J. Roest

**Affiliations:** Utrecht University, Utrecht, the Netherlands (M.A.M. van Dijk, J.A. Wagenaar, E.M. Broens);; Wageningen Bioveterinary Research, Lelystad, the Netherlands (M.Y. Engelsma, M.E. Holtslag, P.T.J. Willemsen, J.A. Wagenaar, H.I.J. Roest);; Netherlands Food and Consumer Product Safety Authority, Utrecht (V.X.N. Visser, M.A.H. Spierenburg)

**Keywords:** *Brucella suis* biovar 1, brucellosis, zoonoses, dog, raw meat diet, foodborne transmission, bacteria, the Netherlands, food safety

## Abstract

A *Brucella suis* biovar 1 infection was diagnosed in a dog without typical exposure risks, but the dog had been fed a raw meat–based diet (hare carcasses imported from Argentina). Track and trace investigations revealed that the most likely source of infection was the dog’s raw meat diet.

Exposure risks for *Brucella suis* infection typically include contact with wildlife or livestock, breeding, and travel to brucellosis-endemic areas. We report a case of *B. suis* infection in a dog for which the risk was determined to be a raw meat–based diet.

## The Case

 In November 2016, a 6-year-old, intact, male American Staffordshire terrier was admitted to a primary care veterinary clinic in the Netherlands, where fever, ascites, and epididymitis/orchitis were detected. Because clinical signs did not improve after a 5-day course of amoxicillin/clavulanic acid (12.5 mg/kg 2×/d), the dog was neutered. During surgery, purulent exudate from the epididymis was noted; this exudate and abdominal fluid were collected and submitted to a routine veterinary diagnostic laboratory. Both samples yielded bacterial growth that was identified by matrix-assisted laser desorption/ionization time-of-flight (MALDI-TOF) mass spectrometry (Bruker Daltonics, Bremen, Germany) as *Brucella* spp. The Dutch National Reference Laboratory identified the isolate by MALDI-TOF mass spectrometry (with an in-house extended database) as *Brucella suis* biovar 1, and the EU reference laboratory confirmed this phenotypically (*1*). One isolate was sequenced and molecularly characterized in silico by multilocus variable-number tandem-repeat analysis (MLVA) as Ms Bruce 06/08/11/12/42/43/45/55/18/19/21/04/07/09/16/30: 2/3/6/10/4/1/5/2/4/38/9.5/5/4/8/5/3 and by multilocus sequence typing (MLST) as sequence type (ST) 14 (*2*–*4*). 

After diagnosis confirmation, serum and urine samples were collected from the dog. Serologic testing for *B. suis* yielded a positive result by microscopic agglutination test (MAT; >120 IU/mL) and rose bengal test (*4*,*5*). Serologic test results for *B. canis* (serum agglutination test <50 IU/mL) (*1*) and bacteriologic culture of a urine sample were negative. Despite treatment with doxycycline (10 mg/kg 1×/d for 14 days starting 3 days after neuter), the dog did not recuperate and because of the poor prognosis was euthanized. Postmortem examination of the dog was performed, and samples from kidney, spleen, prostate, liver, and abdominal lymph nodes were tested by PCR (*4*). Only the prostate yielded a positive result for *Brucella* spp.

Because brucellosis is notifiable in the Netherlands, the Incidence Crisis Centre of the Netherlands Food and Consumer Product Safety Authority was notified. The Centre started investigations to track potential transmission and trace the source of infection. The owners of the index dog were asked to list all dogs that had had frequent contact with their dog during the previous 2–3 months. From the 5 contact dogs identified, blood samples were collected (twice, 4 weeks apart) for serologic testing (MAT and rose bengal) and urine samples were collected for bacteriologic culture. Blood from 1 contact dog yielded a weakly positive result for *B. suis* antibodies (MAT 30 IE/mL; rose bengal negative) at both collection times. An acute infection in this dog was considered unlikely because no seroconversion was detected. All other dogs yielded negative serologic results. All urine samples were bacteriologically negative.

The owners of the index dog reported no relevant exposure risks except that the dog was fed a raw meat–based diet (usually commercial mixed raw feed and in June–July 2016 unprocessed heads of hares, all from the same supplier). Because raw meat consumption has been associated with *B. suis* infections in dogs (*6*,*7*), the feed was considered a potential source of infection. In December 2016, the index dog owner provided leftovers of the commercial mixed raw feed, which we tested by PCR for the presence of porcine DNA and *Brucella* spp.; results for both were negative. The investigators visited the raw feed supplier and sampled a (not yet marketed) 30,000-kg batch of hare carcasses imported from Argentina. Of 40 representative samples, 2 yielded a positive PCR result for *Brucella* spp. and were subsequently cultured. Colonies from 1 sample were confirmed by MALDI-TOF mass spectrometry (with an in-house extended database) to be *B. suis* biovar 1. One isolate was sequenced and molecularly characterized in silico by MLVA and MLST (ST14) (*2*–*4*). The isolates from the index dog and from the batch of hare carcasses showed high similarity (only 1 locus difference in the MLVA Ms07: 4 repeats dog isolate; 6 repeats hare isolate). Similarity with 24 closely related reference isolates from a public database (http://microbesgenotyping.i2bc.paris-saclay.fr/) was much lower (Figure).

**Figure:**
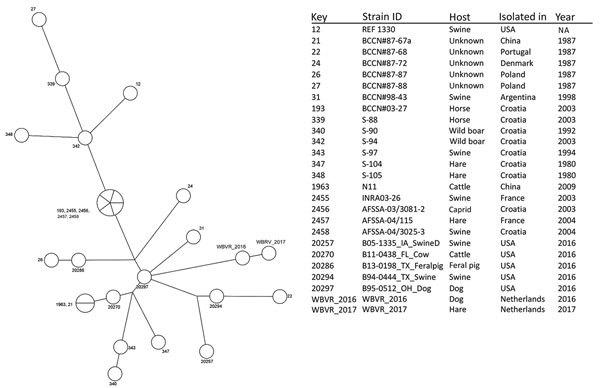
Maximum parsimony analysis on MLVA-16 (multilocus variable-number tandem-repeat analysis) of genotypes from 2 recent *Brucella suis* biovar 1 isolates from the Netherlands (WBVR2016 from a dog and WBVR2017 from hare carcasses) in conjunction with *B. suis* biovar 1 strains of the highest similarity from a public database (http://microbesgenotyping.i2bc.paris-saclay.fr/) with 521 entries of *B. suis*. NA, not available.

## Conclusions

This *B. suis* biovar 1 infection in a dog in the Netherlands was linked to its commercial raw meat–based diet. Canine infections with this biovar have been documented in *B. suis* biovar 1–endemic areas (e.g., Australia and Latin America), mostly associated with exposure to feral pigs or consumption of raw feral pig meat (*6*,*7*). In the case we report, the *B. suis* biovar 1 infection most likely originated from hare carcasses imported from Argentina into the Netherlands. *B. suis* biovar 1 is endemic to Latin America and has been isolated from hares (*7*–*9*). The dog showed clinical signs ≈4 months after it had been fed raw hare heads from a supplier of commercial raw feed. The presence of *B. suis* biovar 1 in another batch of hare carcasses from the same supplier makes foodborne transmission highly likely. The genotypic similarity between the isolates from the dog and the feed and the fact that the supplier imported multiple batches from the same slaughter plant in Argentina during the preceding months confirms the feed as the most probable source of infection.

This report illustrates possible implications of the global trade of raw meat. Importation of hare carcasses, whether or not approved for human consumption, from countries outside the European Union into the European Union is legal. Because the aforementioned batches of hare carcasses from Argentina were approved for human and animal consumption, humans and other animals were potentially at risk when handling or consuming meat products from these batches. 

Medical microbiologists of the Municipal Health Service assessed the zoonotic risks for all persons who had come in contact with the dog or with samples from the dog or hare carcasses. Five laboratory technicians who had been exposed to pure cultures (before bacterial identification) were given postexposure prophylaxis and tested for seroconversion to *B. suis* (postexposure weeks 2, 4, 6, and 24) according to national guidelines (*10*). To our knowledge, no human infections were linked to this case.

*B. suis* biovar 1 is a potential threat to the pig farming industry because introduction of *B. suis* into pig herds can have substantial economic consequences (*11*). A striking detail is that the last *B. suis* infection in pigs in the Netherlands (1969) was associated with swill feeding of hares imported from Argentina (*12*).

In response to our findings, preventive measures were implemented (e.g., sampling of imported raw meat and communication of risk to international authorities and raw-feed suppliers). This case stresses the microbiological risks for humans and animals of feeding raw meat–based diets, which has become increasingly popular among pet owners (*13*). This case also highlights the need for a One Health approach because *B. suis* biovar 1 is a zoonotic agent and can cause severe infections in humans (*14*,*15*).
